# Female genital mutilation and its effects among women of reproductive age in Kenya: Insights from Kenya Demographic and Health Survey 2022

**DOI:** 10.1371/journal.pone.0337399

**Published:** 2025-11-20

**Authors:** Jovinary Adam, Joyce Jebet

**Affiliations:** 1 Jeyibm Investment Tanzania Limited, Dar es Salaam, Tanzania; 2 Aga Khan University - Nairobi, Kenya; Central South University, CHINA

## Abstract

**Introduction:**

Female genital mutilation (FGM) is a significant public health issue rooted in cultural traditions, affecting millions of women and girls. In Kenya, the practice is prevalent in various ethnic groups and is often associated with social and economic pressures.

**Objective:**

To determine the prevalence of FGM, the factors associated with FGM, and its effects among women of reproductive age in Kenya.

**Methods:**

This study used secondary data of a total 16,716 weighted women of reproductive age drawn from the 2022 Kenya Demographic and Health Survey. The study used the “svy” command in Stata to assign the sample weight. Multivariable logistic regression was used to assess significant factors associated FGM and statistical significance was set at a 5% significance level.

**Results:**

The overall prevalence of FGM among women of reproductive age in Kenya was 14.8% (95% CI = 13.98, 15.67). The findings showed that the following factors were associated with FGM: age 30–39 years (aOR=1.66, 95% CI = 1.28, 2.16) and age 40–49 years (aOR=2.71, 95% CI = 2.06, 3.57), residing in rural areas (aOR=1.37, 95% CI = 1.11, 1.68), no education (aOR=3.45, 95% CI = 2.69, 4.41) or primary education (aOR=1.40, 95% CI = 1.20, 1.64), being poor (aOR=1.76, 95% CI = 1.38, 2.25) or middle-income (aOR=1.34, 95% CI = 1.08, 1.66), being married (aOR=1.71, 95% CI = 1.37, 2.13) or separated (aOR=1.56, 95% CI = 1.17, 2.07), believing that FGM is required by religion (aOR=2.03, 95% CI = 1.37, 2.99), culture (aOR=4.79, 95% CI = 3.80–6.05), and society (aOR=2.65, 95% CI = 1.96, 3.58), FGM continued to be practiced (aOR=2.76, 95% CI = 2.14, 3.55), having a male household head (aOR=1.19, 95% CI = 1.02, 1.38), and never listening to the radio (aOR=1.31, 95% CI = 1.11, 1.54). Additionally, the results indicated that the most common side effects were severe pain (70.3%) and heavy bleeding (45.9%).

**Conclusion:**

FGM is still a prevalent practice in Kenya despite being outlawed. gender and social norms contribute to the sustenance of the practice. The emerging trends including medicalization and change in the age of cutting need to be addressed. The factors that accelerate and enhance the practice of FGM need to be addressed and advocate more against the practice of FGM as it is a violation of human rights.

## Background

FGM is a global concern. To date, the practice is reported in 30 countries in Africa and in a few countries in Asia and the Middle East. Some forms of FGM are also reported to occur among certain ethnic groups in Central and South America, and Eastern Europe [1].

Over 230 million girls and women worldwide have undergone FGM. Africa accounts for the largest share of this total, with over 144 million. Asia follows with over 80 million, and a further 6 million are in the Middle East. Another 1–2 million are affected in small practicing communities and destination countries for migration in the rest of the world [2].

The procedure of FGM has been practiced in Kenya for centuries among a number of ethnicities, including the Somali, Kisii, Embu, Kalenjin, Kamba, Kikuyu, Taita/Taveta, Maasai, Meru [3–6]. The reasons for performing FGM vary from culture to culture and region to region. Despite the fact that FGM is a violation of human rights, this practice has been upheld with men propagating the practice in many cultures. Several efforts have been made to eradicate the practice, however, it is deeply rooted in many cultures [7]. It has also been established that religious land political leaders have enabled the sustenance of the practice in the communities in addition to poor enforcement of the law [8]. FGM in Kenya is deeply rooted in cultural traditions related to heritage and the transition to adulthood [8]. It is motivated by beliefs about appropriate sexual behavior for women and preparation for marriage. Many communities view female external genitalia as unclean, and its removal is seen as a way to promote hygiene and aesthetic appeal [9]. FGM, especially clitoral removal, is thought to reduce female sexual desire, uphold chastity, enhance male pleasure, and prevent issues like body odor and promiscuity [10]. FGM is a source of income for practitioners who are paid a certain amount of money for performing the operation and material incentives to the girls, which include new clothes, shoes, money and other related gifts [8,11].

Studies conducted in Kenya reported that girls are not allowed to have any autonomy over their own bodies. Most girls are not involved in decision making concerning circumcision. Mothers, aunts, grandmothers and other relatives were the ones who decided whether the girls should or should not be circumcised [11,12]. With the emerging trends in the practice of FGM, where people hide to carry out the practice, medicalization has been found to be one of the factors contributing to the high prevalence [13].

Female genital mutilation is detrimental to the physical, social, and emotional well-being of women and girls, and it has the potential to cause serious medical complications. Beyond excruciating pain and severe bleeding, long-term physical and psychological damage can result from the procedure, including infection, infertility and post-traumatic stress disorder. Many girls and women who have been cut face reproductive challenges, including newborn death, stillbirth, and postpartum haemorrhage [2,14–16].

The government of Kenya has taken steps in the campaign against FGM by enacting laws and establishment of the Anti-FGM Board whose mandate includes designing, supervising and coordination of all programmes aimed at the eradication of FGM in the country. The Board has made significant progress in the anti-FGM campaign which needs to be sustained [17]. Despite prohibition of FGM, is still being practiced in Kenya.

Previous studies reported that FGM have been associated with education, economic status, place of residence, age, employment, ethnicity and marital status [8,18–21]. Furthermore, previous studies conducted in Kenya focused on FGM in specific areas, such as counties [8,22]. However, this study uses a national and large-scale data set. Therefore, this study aimed to determine the prevalence of FGM, the factors associated with FGM, and its effects among women of reproductive age in Kenya using Kenya Demographic and Health Survey 2022.

## Methods

### Data source

The analysis utilized the latest nationally representative data from the 2022 Kenya Demographic and Health Survey. Conducted between 17 February to 31 July 2022, implemented by Kenya National Bureau of Statistics (KNBS) in collaboration with the Ministry of Health (MoH) and other stakeholders. It was funded by the United States Agency for International Development (USAID) that offers financial support and technical assistance for population and health surveys in countries worldwide. The 2022 Kenya DHS survey collected data from a nationally representative probability sample of households, women of reproductive age, and men in the sampled households [23].

### Study design

This study was a cross-sectional, analysis of secondary data from the Kenya Demographic and Health Survey 2022. The sample design was carried out in two stages and intended to provide estimates for the entire country for urban and rural areas. The sample for the 2022 KDHS was drawn from the Kenya Household Master Sample Frame (K-HMSF). The 2022 KDHS employed a two-stage stratified sample design, where in the first stage 1,692 clusters were included. Household listing was carried out in all the selected clusters, and the resulting list of households served as a sampling frame for the second stage of selection, where 25 households were selected from each cluster. However, after the household listing procedure, it was found that some clusters had fewer than 25 households; therefore, all households from these clusters were selected into the sample. The individual record (IR) file from the women’s data set for this study was used. The unit of analysis for this study was women aged 15–49, who amounted to 16,716 [23].

### Study variables

#### Outcome variable.

The study’s outcome variable was FGM among women aged 15–49 years. The respondents were asked: ““Have you yourself ever been circumcised?”. The response was binary with “Yes” coded as ‘1’denoting “have circumcised” and “No “coded as ‘0’denoting “have never circumcised”. To ensure data integrity, incomplete or missing responses related to FGM status were excluded from the analysis.

#### Exposure variable.

The explanatory variables were chosen based on their availability within the data set and on the related relevant literature. Thus, they included age, place of residence, education level, wealth status, marital status, sex of household head, listening radio, watching television, FGM is required by region, FGM is required by society, FGM is required by culture, and FGM continuing or stopped [18,19,21,24,25]

These variables were further categorized into covariates such as age (in years) (15–19, 20–29, 30–39, 40–49), place of residence (urban, rural), education level (no education, primary, secondary and above) wealth status (poor, middle, rich), marital status (never in union, married, separated), sex of household head (male, female), listening radio (yes, no), watching television (yes, no), FGM is required by region (yes, no), FGM is required by society (yes, no), FGM is required by culture (yes, no), and FGM continuing or stopped (continue, stopped).

Moreover, age (in years) variable was originally divided into seven categories: 15–19, 20–24, 25–29, 30–34, 35–39, 40–44, and 45–49. However, it was recoded into four categories: 15–19 and 20–29, 30–39, 40–49. The wealth index variable was originally divided into five categories: poorest, poorer, middle, richer, and richest. However, it was recategorized into three groups for a more streamlined analysis: combining ‘poorest’ and ‘poorer’ into the ‘poor’ category, ‘richer’ and ‘richest’ into the ‘rich’ category, and retaining ‘middle’ as a separate third category [26]. Additionally, the employment status variable was originally divided into eleven categories: not working, professional/technical/managerial, clerical, sales, agricultural (self-employed), agricultural (employee), household and domestic, services, skilled manual, unskilled manual, and other. For analysis, it was recoded into two categories: ‘unemployed’ for those not working, and ‘employed’ for all other categories. Moreover, marital status was originally divided into six categories: never in union, married, living with partner, widowed, divorced, and no longer living together/separated. However, it was recoded into three categories for analysis: retaining the ‘never in union’ category, combining ‘married’ and ‘living with partner’ into the ‘married’ category, and combining ‘widowed,’ ‘divorced,’ and ‘no longer living together/separated’ into the ‘separated’ category [24].

Education level was divided into no education, primary, secondary and higher. However, it was recoded into three categories where secondary education and higher were combined into secondary and above while no education and primary were retained [27]. Furthermore, listening to the radio and watching television were previously divided into three categories: at least once a week, less than once a week, and not at all. However, these were recategorized into two categories: ‘yes’ for at least once a week and less than once a week, and ‘no’ for not at all. Additionally, FGM, as required by culture, society, or religion, was previously categorized in the DHS into three options: ‘no,’ ‘yes,’ and ‘don’t know.’ However, these were recategorized into two categories: ‘yes’ (for ‘yes’) and ‘no’ (where ‘don’t know’ was recoded as ‘no’) [28]. Other variables, including place of residence (urban, rural), Sex of household head (male, female), were utilized as recorded in the DHS dataset.

### Statistical analysis

The study employed Stata version 18 to perform data analyses. Descriptive analysis was used by calculating weighted frequencies and percentages, through which prevalence of FGM obtained and described the background characteristics of the study participants. The “svy” command was used in STATA for assigning the sample weight and to adjust for the clustering effect and sample stratification. The chi-square test was used to examine the association between the outcome variable (female genital mutilation) and categorical independent variables. All variables with a p-value < 0.2 in the bivariate analyses were included in the multivariable logistic regression model. Variables with p-values less than 0.05 were considered to be independently significant associated with the FGM. The strength of the association was assessed using the adjusted odds ratio (aOR) along with its corresponding 95% confidence interval (CI).

### Ethics approval and consent to participate

DHS surveys are conducted incompliance with Helsinki Declaration for biomedical research. The study analysed the collected data from the Kenya Demographic and Health Survey, which had already obtained ethical clearance from the Kenya National Bureau of Statistics (KNBS) for data collection; hence, this study did not need additional ethical clearance. However, permission to use the data were requested from the DHS custodian USAID MEASURES.

## Results

### Background characteristics of the study participants

The analysis for this study involved a sample of 16,716 women of reproductive age (15–49 years) in Kenya. A significant portion of the participants were aged 20–29 years (35.8%). Over half of the participants lived in rural areas (59%). The majority had secondary education or higher (58%), while 5.5% reported had no education. Nearly half (48.2%) were classified in the rich wealth quintile. More than half (55.7%) were married. Ninety-six percent of women do not believe that their religion requires FGM. Moreover, 88% of women believed that their culture does not require FGM, and 92% of women do not believe FGM is required by their society. Other characteristics of the study participants are presented in Table 1.

**Table 1:** Background characteristics of the study participants and their association with FGM among women of reproductive age in Kenya (n = 16,716) (Weighted sample).

Variable	Frequency	Percentage	Number (%) ever circumcised	P-value
**Age group (years)**				
15-19	3,125	18.7	286(9.1)	**<0.001**
20-29	5,979	35.8	687 (11.5)	
30-39	4,652	27.8	807 (17.4)	
40-49	2961	17.7	696 (23.5)	
**Place of residence**				
Urban	6,850	41.0	662(9.7)	**<0.001**
Rural	9,866	59.0	1,814 (18.4)	
**Education level**				
Secondary+	9,689	58.0	832(8.6)	**<0.001**
Primary	6,107	36.5	1,126 (18.4)	
No education	920	5.5	518(56.3)	
**Wealth status**				
Rich	8,057	48.2	696 (8.6)	**<0.001**
Middle	3,086	18.5	450 (14.6)	
Poor	5, 573	33.3	1,330 (23.9)	
**Employment status**				0.0875
Employed	10,012	59.9	1,429 (14.3)	
Unemployed	6,704	40.1	1,047 (15.6)	
**Marital status**				
Never in union	5,345	32.0	401 (7.5)	**<0.001**
Married	9,318	55.7	1,739 (18.7)	
Separated	2,054	12.3	336 (16.3)	
**FGM is required by religion**				
No	15,584	96.1	2,018 (13)	**<0.001**
Yes	621	3.9	458 (73.1)	
**FGM continue or stopped**				
Stopped	15,212	93.8	1,824 (12)	**<0.001**
Continued	993	6.2	652 (65.4)	
**FGM is required by culture**				
No	14,308	88.3	1,375 (9.6)	**<0.001**
Yes	1,897	11.7	1,100 (57.9)	
**Sex of household head**				
Female	6,569	39.3	854 (13)	**<0.001**
Male	10,152	60.7	1,622 (16)	
**FGM is required by society**				
No	14,947	92.2	1,633 (10.9)	**<0.001**
Yes	1,258	7.8	842 (66.7)	
**Listening radio**				
Yes	12,621	75.5	1,573 (12.5)	**<0.001**
No	4,095	24.5	903 (22)	
**Watching television**				
Yes	11,046	66.1	1,151 (10.4)	**<0.001**
No	5,670	33.9	1,325 (23.4)	

### The prevalence of FGM among women of reproductive age (15–49 years) in Kenya

**Fig 1:**
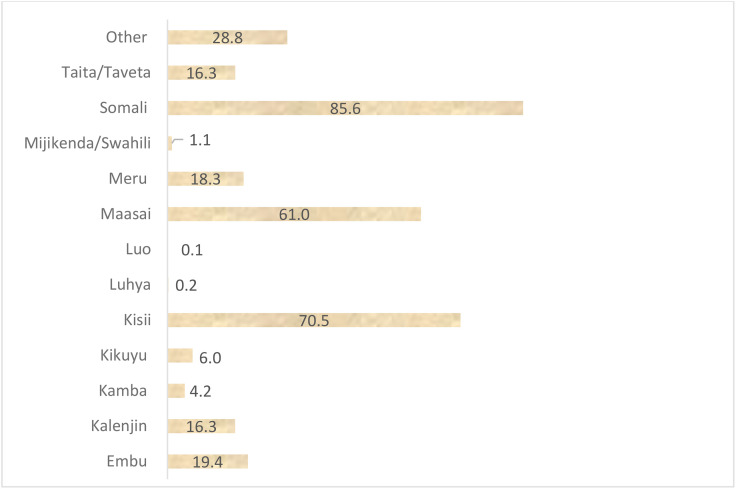
The prevalence of female genital mutilation among women of reproductive age across ethnicity groups in Kenya (%).

**Fig 2:**
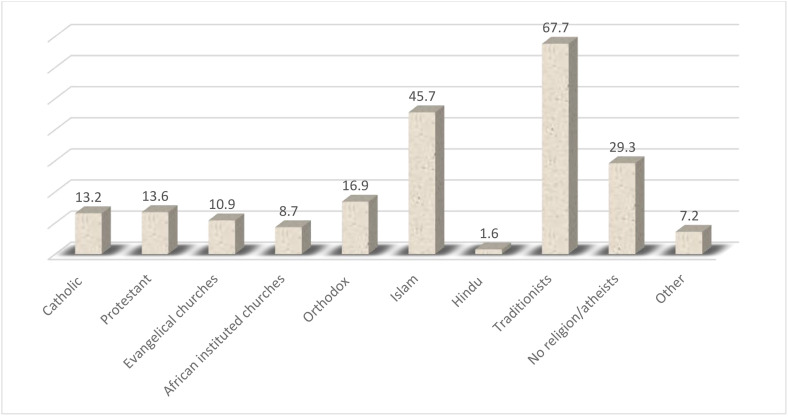
The prevalence of female genital mutilation among women of reproductive age across religion (%).

**Fig 3:**
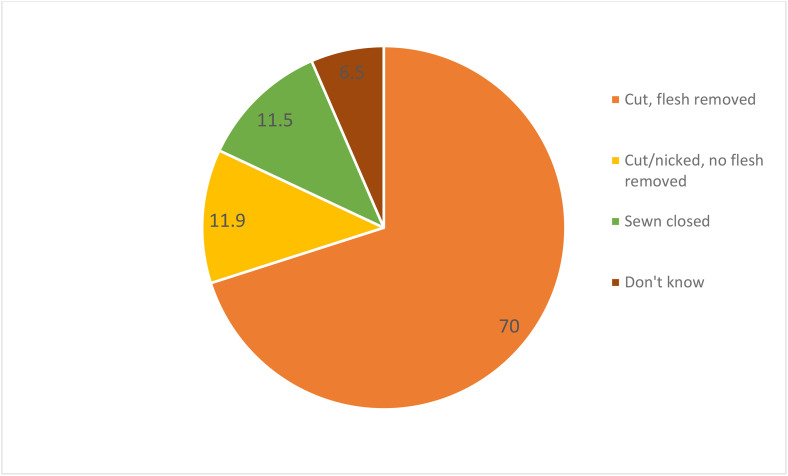
Prevalence of female genital mutilation by type among women age 15-49 years (%).

**Fig 4:**
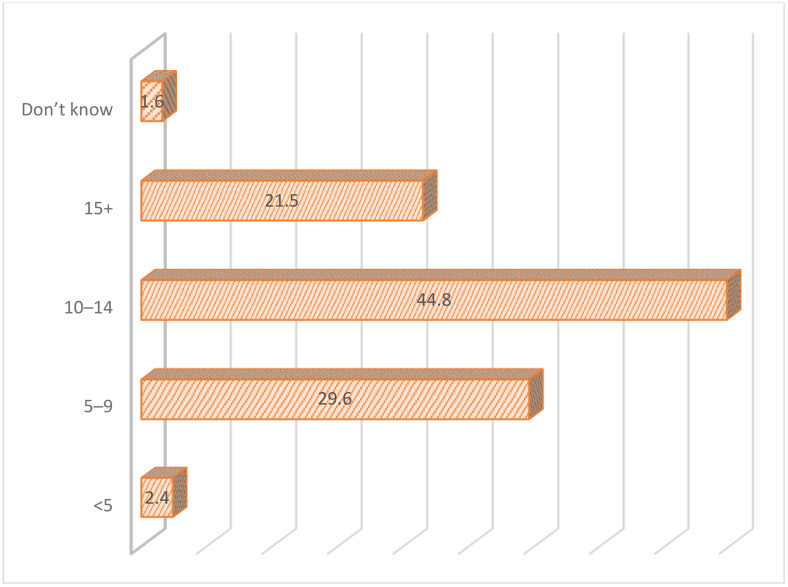
Age at Circumcision: Distribution of women who are circumcised (15–49 years) (%).

The overall prevalence of FGM among women of reproductive age in Kenya was 14.8% (95% CI = 13.98, 15.67). The prevalence of FGM among women of reproductive age in Kenya varied significantly across the ethnicity groups, ranging from <1% among Luo and Luhya to 85.6% among Somali (Fig 1). Additionally, the prevalence of FGM varies across religion in Kenya, ranging from 1.6% among Hindu to 67.7% among traditionists as shown in Fig 2. The most common type of FGM in Kenya is Type II (cut, flesh removed), with almost 70% circumcised women undergoing this procedure (Fig 3). Among circumcised women, majority (45%) were circumcised at age 10–14 years, and only 2% of women age 15–29 years were circumcised when they were under age 5 years (Fig 4).

### Factors associated with FGM among women of reproductive age in Kenya

As shown in the logistic regression results in Table 2, women aged 30–39 years (aOR=1.66, 95% CI = 1.28, 2.16), and 40–49 years (aOR=2.71, 95% CI = 2.06, 3.57) had higher odds of experiencing FGM. Women residing in rural areas had higher odds, (aOR=1.37, 95% CI = 1.11, 1.68), of experiencing FGM than women residing in urban areas. Women with no education (aOR=3.45, 95% CI = 2.69, 4.41), and primary education (aOR=1.40, 95% CI = 1.20, 1.64), had higher odds of being circumcised than those with secondary education and above. Moreover, the odds of being circumcised were higher for women in the poor quintile (aOR=1.76, 95% CI = 1.38, 2.25) and middle quartile (aOR=1.34, 95% CI = 1.08, 1.66) than their counterparts in rich quintile. Married women (aOR=1.71, 95% CI = 1.37, 2.13) and separated women (aOR=1.56, 95% CI = 1.17, 2.07) had significantly higher odds of being circumcised than women who were never in union. Women who said FGM is required by religion (aOR=2.03, 95% CI = 1.37, 2.99), culture (aOR=4.79, 95% CI = 3.80, 6.05), and society (aOR=2.65, 95% CI = 1.96, 3.58) had higher odds of being circumcised. Additionally, women who needed FGM to continue (aOR=2.76, 95% CI = 2.14, 3.55), male household (aOR=1.19, 95% CI = 1.02, 1.38), women who were not listening radio (aOR=1.31, 95% CI = 1.11, 1.54) had higher odds of being circumcised.

**Table 2:** Logistic regression results on the factors associated FGM among women of reproductive age (15-49 years) in Kenya.

Variable	OR	95% CI	P- value	aOR	95% CI	P- value
**Age group (years)**						
15-19	**Ref**			**Ref**		
20-29	1.29	1.08 - 1.53	**0.004**	1.23	0.97 - 1.56	0.088
30-39	2.09	1.76 - 2.48	**<0.001**	1.66	1.28 - 2.16	**<0.001**
40-49	3.06	2.55 - 3.68	**<0.001**	2.71	2.06 - 3.57	**<0.001**
**Place of residence**						
Urban	**Ref**			**Ref**		
Rural	2.11	1.81 - 2.47	**<0.001**	1.37	1.11 - 1.68	**0.003**
**Education level**						
Secondary+	**Ref**			**Ref**		
Primary	2.41	2.10 - 2.77	**<0.001**	1.40	1.20 - 1.64	**<0.001**
No education	13.71	11.44 - 16.44	**<0.001**	3.45	2.69 - 4.41	**<0.001**
**Wealth status**						
Rich	**Ref**			**Ref**		
Middle	1.81	1.52 - 2.15	**<0.001**	1.34	1.08 - 1.66	**0.008**
Poor	3.32	2.81 - 3.92	**<0.001**	1.76	1.38 - 2.25	**<0.001**
**Employment status**						
Employed	**Ref**			**Ref**		
Unemployed	1.11	0.98 - 1.25	0.088	0.91	0.78 - 1.06	0.211
**Marital status**						
Never in union	**Ref**			**Ref**		
Married	2.83	2.43 - 3.28	**<0.001**	1.71	1.37 - 2.13	**<0.001**
Separated	2.41	1.95 - 2.97	**<0.001**	1.56	1.17 - 2.07	**0.002**
**FGM is required by religion**						
No	**Ref**			**Ref**		
Yes	18.24	14.06 - 23.66	**<0.001**	2.03	1.37 - 2.99	0.812
**FGM continue or stopped**						
Stopped	**Ref**			**Ref**		
Continued	13.85	11.42 - 16.80	**<0.001**	2.76	2.14 - 3.55	**<0.001**
**FGM is required by culture**						
No	**Ref**			**Ref**		
Yes	12.92	11.16 - 14.95	**<0.001**	4.79	3.80 - 6.05	**<0.001**
**Sex of household head**						
Female	**Ref**			**Ref**		
Male	1.27	1.13 - 1.43	**<0.001**	1.19	1.02 - 1.38	**0.024**
**FGM is required by society**						
No	**Ref**			**Ref**		
Yes	16.36	13.61 - 19.65	**<0.001**	2.65	1.96 - 3.58	**<0.001**
**Listening radio**						
Yes	**Ref**			**Ref**		
No	1.99	1.76 - 2.24	**<0.001**	1.31	1.11 - 1.54	**0.011**
**Watching television**						
Yes	**Ref**			**Ref**		
No	2.62	2.32 - 2.97	**<0.001**	1.14	0.97 - 1.33	0.114

### The effects of FGM among women of reproductive age in Kenya

**Fig 5:**
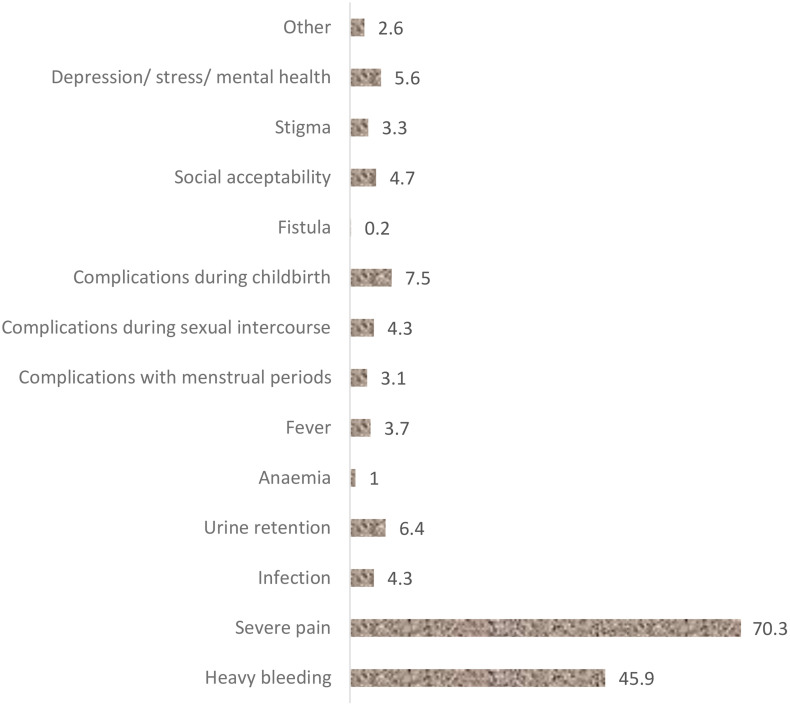
The effects of female genital mutilation among women of reproductive age in Kenya (%).

Among circumcised women, 772 (31.2%) experienced side effects from undergoing circumcision. The most common side effects were severe pain (70.3%) and heavy bleeding (45.9%) as shown in Fig 5.

## Discussion

The overall prevalence of FGM among women of reproductive age in Kenya was 14.8%. The study revealed that most common type of FGM in Kenya is Type II. The practice was mostly found among the Somali, Kisii and Maasai. The study identified several factors associated with FGM. Among these factors, the study identified age, education level, marital status, wealth index, FGM required by religion, FGM required by society, FGM required by culture, FGM continued to be practiced, sex of household head, and listening to radio. The study found that most common side effects were severe pain and heavy bleeding.

The prevalence of FGM varied across the counties by ethnicity, religion, type of the cut and age of cutting. FGM is a social phenomenon that is deeply rooted in many African cultures, making it more prevalent in the Africa region [29]. In this study, the most common type of cut was type II, where the women’s clitoris is cut and flesh is removed, with 70% of women having undergone this cut. This could be attributed to the ethnicity practices on the cutting, where FGM is viewed as a social norm, thus one feels acceptable after undergoing the cut. There has been a change in the cutting where the communities that used to practice severe types are now moving to less severe types with the view of sustaining their practice.

The practice was mostly found among the Somali, Kisii and Maasai. In this communities, the practice is deeply entrenched in culture and religion. A study of the Somali refugee community in eastern Ethiopia revealed that the commonest types of cut was clitoral cutting and narrowing of the vagina, indicating a shift from the more severe types to the milder type [30]. The prevalence of FGM among various religions was highest among the traditionalists, those who subscribe to cultural practices followed by Islam. The findings are similar to an Ethiopian study, which established women were reluctant to end the practice since it was their norm [31]. FGM is deeply rooted in culture, thus the traditionalists seek to uphold their practice [32], while the Islam justify the practice indicating they are following the teaching of Sunna practice [33]. Contrary to the belief, there is doubt on the authenticity of the narratives and the Quran does not make any reference to FGM [34].

Age is an important factor in determining the practice of FGM. This study established that most of the women who had undergone FGM were aged between 30–49 years old. Older women were likely to have undergone FGM practice since this was the norm, however with more understanding of the effects, older women are unlikely to allow their girls to undergo the practice. This explains the difference in the ages where there are more older women, above 30 years who have undergone FGM compared to the younger women. However, it should be noted that the age of cutting is likely to be a younger age. In the Gambia, five out of ten girls aged 0–15 years have undergone FGM [35]. The World Health Organization (WHO) has established that the average age of cutting is between infancy and 15 years old [7]. Contrary findings were established in a study in Mali which noted that the odds of cutting increased with age [36]. Although most communities carried out the practice during puberty, there is a current trend where communities are reverting to an early age where the girls are less enlightened about the practice.

The current study established that the level of education was significantly associated with undergoing FGM practice. Similar findings have established that in communities with high literacy levels and low community prevalence, the likelihood of FGM is lower [37,38]. Education creates awareness and empowerment, thus women who are educated are likely to say no to FGM because they understand the effects as compared to their counterparts who are likely to undergo the practice. Women with no education had higher odds of undergoing FGM as compared to those who are more educated. In a study of Sierra Leone Demographic Heath Survey, it was established that women of higher education level were likely to advocate for FGM to be discontinued [19]. Similar findings have been established in other studies [39–41]. On the other hand, FGM influences a girl’s participation in education. In many communities, once a girl undergoes FGM, they are likely to be married off, thus fail to continue with education. This will have a ripple effect on the girl’s life as it is likely to contribute to lack of empowerment and contribute to economical disempowerment [42–44].

The findings of the current study revealed that the respondents believed that FGM was required by religion. Similar findings have been established by an Ethiopian study which reported that Christian religion was significantly associated with FGM [31]. This could be attributed to in-built religious teachings. Similarly, a systematic review established that parents and religious leaders enforce the tradition to sustain their cultural practices [16]. Religion has influenced the practice of FGM, with the argument that it makes one clean and acceptable to the Creator. Religion and culture are key facilitators of FGM owing to the perception and beliefs about the same [36,45,46]. It has also been established that religious and political leaders have enabled the sustenance of the practice in the communities in addition to poor enforcement of the law [8].

FGM is viewed as a social norm, making it socially acceptable, thus women tend to undergo the practice to be acceptable in their communities. Similar findings have been established in a study examining Chad demographic and health survey data [36]. A lower level of education and Muslim religion has been associated with FGM [38], which is similar to the findings of the current study.

The marital status is also an influencing factor. The current study established that married women had higher odds of being circumcised compared to women who were never in a union. This could be attributed to FGM being viewed as a rite of passage and for a woman to get married, they need to undergo the practice in many communities [47]. Similar findings were established in a Ghanaian study which reported that junior wives entering a polygamous relationship were more likely to be circumcised [47]. In addition, in many settings, married women often are unlikely to make decisions being in patriarchal societies. The decision of undergoing FGM, being a social norm is mostly undertaken by men, thus are likely to be on the affirmative of allowing their girls to undergo the practice [41,48].

Consistent with studies conducted in Tanzania and Ethiopia [24,25,27], women residing in rural areas were more likely to undergo the practice. This could be attributed to the cultural norms being held highly in the rural areas compared to the urban settings where there is a mixture of populations and possibility of eroded cultures. In the emerging trends though, the practice is now being moved to urban settings and often done in secrecy.

Regarding the wealth status, those from poorer backgrounds were more likely to undergo FGM compared to the rich. Poverty has been found to be one of the drivers of FGM [49,50]. Women of low social economic status are likely to have low levels of education, thus are also likely to be less empowered.

The study found that culture had significantly associated with FGM. The possible explanation could be FGM is s viewed as a traditional practice that has been passed down through generations, making it difficult to challenge or change. In most of the cultures, it was reported that FGM has stopped, however it is important to note that FGM still exists in varied amounts in various communities. The current results illustrate existence of FGM in various Counties ranging from 0.1% in the traditionally non-practicing communities to 86.5% in the practicing communities. The current results also highlight a high prevalence of FGM in Kisii County, which is one of the Counties with high prevalence of medicalization of FGM. There has been a growing trend in medicalization of FGM. In Kenya, Kisii, located in south-western region is one of the Counties with high cases of medicalization [13]. FGM is used as a symbol of ethnic identity [8,51]

The main stream media and social media are important tools in influencing and disseminating information against FGM. This could be done through featuring stories, documentaries and hashtags among others. Regarding mass media, listening to the radio and watching television were significantly associated with FGM. Media is one of the important technologies in creating awareness. Similarly in Tanzania, watching television at least once a week was associated with decreasing incidence of FGM among adolescent girls [24]. A case study conducted in Western Kenya illustrated that the media is powerful and can be used to change gender and social norms through locally produced content [52].

FGM affects women and girls physically, psychologically, emotionally, sexually and mentally [7]. It is a social consequence related to gender and social norms [53]. In the current study, the most reported complications were the short-term complications including severe pain and bleeding. However, the long-term complications were also reported. In many instances, the short-term complications are likely to be reported owing to their severity and the risk of losing life. However, the long-term complications are likely to be unreported owing to the stigma associated with them. As a result, many women would live with these complications.

Complications of childbirth have been highlighted in the current study. This is linked to the scar tissue forming on the pelvic outlet thus contributing to obstructed labour. In women who have undergone infibulation, the consequences are severe in that they would need to be de-infibulated before penetration during sexual intercourse, and during birth, there is a risk of bilateral episiotomy to open the perineum and allow the baby to pass through [16]. Similar findings were established in a study in Narok County, Kenya, which reported that women who have undergone FGM experience obstetric effects including obstructed labour, perineal tears and postpartum haemorrhage [54].

Mental and sexual health complications have been reported as effects of FGM. Although the burden of mental illnesses and sexual complications in the current study seems low, 5.6% and 4.3% respectively, many girls and women have experienced stigma in reporting these issues. In a scoping review on African population of women who had undergone FGM, the study established that there was a clear distinction of the sexual functioning of those who had and not undergone FGM [15]. In addition, women who previously experienced sexual intercourse before the cut and after the cut reported that ‘the heat goes away’, meaning they never enjoyed sexual intercourse as they did previously [55]. Women who had experienced FGM on the Female Sexual Functioning Index had reduced scores in all domains [14]. Regarding mental health, many women experienced post-traumatic stress disorder, anxiety and depression following the cut [15].

### Study strength and limitations

The strength of this study lies in its use of nationally representative data. This large sample size facilitates the generation of national estimates, thereby enhancing the generalizability of the findings and offering a thorough understanding of FGM across various socio-demographic groups. Additionally, the application of sample-weighted data ensures that the results accurately represent the broader population, minimizing potential biases that could occur with unweighted analyses.

The findings of the survey are based on self-reporting, as such there could be under or misreporting. There might have been recall bias especially when interviewing on the types of FGM, as such leading to inaccuracies of data. This being a survey, there was a limitation of causal inference. Intra-ethnic variations were not captured in the study, and this may influence reporting of prevalence. There was a limitation in the exploration of the drivers of FGM as there was no in-depth analysis to explore the social, cultural determinants. Since FGM is illegal in Kenya, some participants may have concealed information fearing repercussions, thus leading to data inaccuracy.

## Conclusion

In Kenya, the practice of FGM still exists, although it is declining. The prevalence, type of cut and age of cutting varies in communities. An emerging trend on the change in age of cutting and medicalization of the practice has been observed. Despite the practice being declared illegal, it is still ongoing with many factors enabling its sustainability. FGM has severe consequences to girls and women ranging from physical, social, psychological and emotional.

### Recommendations

There is need to advocate more against the practice of FGM as it is a violation of human rights. The factors that accelerate and enhance the practice of FGM need to be addressed. The emerging trends including medicalization and change in the age of cutting need to be addressed.

## Abbreviations:

aOR: Adjusted Odds Ratio
CI: Confidence Interval
DHS: Demographic and Health Survey
FGM: Female Genital Mutilation
IR: Individual Record
K-HMSF: Kenya Household Master Sample Frame
KNBS: Kenya National Bureau of Statistics
MoH: Ministry of Health
UNFPA: United Nations Population Fund
UNICEF: United Nations Children Education Fund
VIF: Variance Inflation Factor
WHO: World Health Organization

## References

[1] WHO. WHO guidelines on the management of health complications from female genital mutilation. 2016. 27359024

[2] United Nations Children’s Fund. Female genital mutilation. A global concern 2024 update. New York: UNICEF. 2024. https://www.unicef.org/

[3] United Nations Children’s Fund. A profile of female genital mutilation in Kenya. New York: United Nations University Press. 2020.

[4] Mukabi F, Chepngeno V, Onchagwa D. The “uncut” stigma: Debunking the myths and misconceptions that promote female genital mutilation in Kenya. Int J Acad Res Bus Soc Sci. 2022;12(7):1978–95.

[5] Muhula S, Mveyange A, Oti SO, Bande M, Kayiaa H, Leshore C, et al. The impact of community led alternative rite of passage on eradication of female genital mutilation/cutting in Kajiado County, Kenya: A quasi-experimental study. PLoS One. 2021;16(4):e0249662. doi: 10.1371/journal.pone.0249662 33909635 PMC8081212

[6] Kandala N-B, Nnanatu CC, Atilola G, Komba P, Mavatikua L, Moore Z, et al. A Spatial Analysis of the Prevalence of Female Genital Mutilation/Cutting among 0-14-Year-Old Girls in Kenya. Int J Environ Res Public Health. 2019;16(21):4155. doi: 10.3390/ijerph16214155 31661902 PMC6862646

[7] WHO. Female genital mutilation. 2024.

[8] Sheikh MM, Cheptum JJ, Mageto IG. Factors Linked to Female Genital Mutilation Practice Among Women Living In Alungu Village of Mandera County, Kenya. East Afr Health Res J. 2023;7(1):109–15. doi: 10.24248/eahrj.v7i1.716 37529498 PMC10388673

[9] Kakal T, Hidayana I, Kassegne AB, Gitau T, Kok M, van der Kwaak A. What makes a woman? Understanding the reasons for and circumstances of female genital mutilation/cutting in Indonesia, Ethiopia and Kenya. Cult Health Sex. 2023;25(7):897–913. doi: 10.1080/13691058.2022.2106584 36036163

[10] Oyefara JL. Female genital mutilation (FGM) and theory of promiscuity: Myths, realities and prospects for change in Oworonshoki community, Lagos State, Nigeria. Genus. 2014;70(2–3):7–33.

[11] Ondiek CA. The persistence of female genital mutilation (FGM) and its impact on women’s access to education and empowerment: a study of Kuria district, Nyanza Province, Kenya. University of South Africa. 2010. https://uir.unisa.ac.za/handle/10500/4121

[12] Kusters L, Kok M, Van der Kwaak A. A baseline study on child marriage, teenage pregnancy and female genital mutilation/ cutting in Kenya. J Adolesc Heal. 2017;2(2):41–7.

[13] Kimani S, Kabiru CW, Muteshi J, Guyo J. Female genital mutilation/cutting: Emerging factors sustaining medicalization related changes in selected Kenyan communities. PLoS One. 2020;15(3):e0228410. doi: 10.1371/journal.pone.0228410 32119680 PMC7051066

[14] Ismail SA, Abbas AM, Habib D, Morsy H, Saleh MA, Bahloul M. Effect of female genital mutilation/cutting; types I and II on sexual function: case-controlled study. Reprod Health. 2017;14(1):108. doi: 10.1186/s12978-017-0371-9 28854947 PMC5577780

[15] Tammary E, Manasi K. Mental and sexual health outcomes associated with FGM/C in Africa: a systematic narrative synthesis. EClinicalMedicine. 2023;56:101813. doi: 10.1016/j.eclinm.2022.101813 36880050 PMC9985012

[16] Klein E, Helzner E, Shayowitz M, Kohlhoff S, Smith-Norowitz TA. Female Genital Mutilation: Health Consequences and Complications-A Short Literature Review. Obstet Gynecol Int. 2018;2018:7365715. doi: 10.1155/2018/7365715 30116269 PMC6079349

[17] Republic of Kenya. National policy for the eradication of female genital mutilation: Towards a society free from harmful cultural practices. 2019. https://gender.go.ke/wp-content/uploads/2019/10/NATIONAL-POLICY-FOR-THE-ERADICATION-OF-FEMALE-GENITAL-MUTILATION-.pdf

[18] Ackah JA, Ayerakwah PA, Boakye K, Owusu BA, Bediako VB, Gyesi M, et al. Circumcising daughters in Nigeria: To what extent does education influence mothers’ FGM/C continuation attitudes?. PLOS Glob Public Health. 2022;2(11):e0000660. doi: 10.1371/journal.pgph.0000660 36962539 PMC10021453

[19] Ameyaw EK, Tetteh JK, Armah-Ansah EK, Aduo-Adjei K, Sena-Iddrisu A. Female genital mutilation/cutting in Sierra Leone: are educated women intending to circumcise their daughters?. BMC Int Health Hum Rights. 2020;20(1):19. doi: 10.1186/s12914-020-00240-0 32703226 PMC7376916

[20] Mwanja CH, Herman PZ, Millanzi WC. Prevalence, knowledge, attitude, motivators and intentional practice of female genital mutilation among women of reproductive age: a community-based analytical cross-sectional study in Tanzania. BMC Womens Health. 2023;23(1):226. doi: 10.1186/s12905-023-02356-6 37138247 PMC10158332

[21] Gebeyehu AA, Anteneh RM, Dessie AM, Yenew C. Prevalence and determinants of female genital amputation among adolescent girls and young women in Ethiopia: multilevel analysis. J Health Popul Nutr. 2023;42(1):144. doi: 10.1186/s41043-023-00484-1 38102635 PMC10725002

[22] Everline M. Factors influencing the practice of female genital mutilation in Kenya: a case study of Gachuba division, Nyamira County. University of Nairobi. 2014.

[23] Kenya National Bureau of Statistics KNBS, ICF. Kenya Demographic and Health Survey 2022. Nairobi, Kenya, and Rockville, Maryland, USA: KNBS and ICF. 2023.

[24] Adam J, Charles P. Female genital mutilation and its associated factors among adolescent girls and young women in Tanzania: analysis of the 2022 Tanzania Demographic and Health Survey and Malaria Indicator Survey (2022 TDHS-MIS). BMC Public Health. 2024;24(1):2009. doi: 10.1186/s12889-024-19151-z 39068386 PMC11282644

[25] Nyamhanga T, Kapinga O, Muro BA, Luoga P. Factors associated with female genital mutilation/cutting in Tanzania: insights from Tanzania demographic and health survey 2022. BMC Womens Health. 2025;25(1):415. doi: 10.1186/s12905-025-03965-z 40885968 PMC12398043

[26] Sakeah E, Debpuur C, Oduro AR, Welaga P, Aborigo R, Sakeah JK, et al. Prevalence and factors associated with female genital mutilation among women of reproductive age in the Bawku municipality and Pusiga District of northern Ghana. BMC Womens Health. 2018;18(1):150. doi: 10.1186/s12905-018-0643-8 30227845 PMC6145319

[27] Geremew TT, Azage M, Mengesha EW. Hotspots of female genital mutilation/cutting and associated factors among girls in Ethiopia: a spatial and multilevel analysis. BMC Public Health. 2021;21(1):186. doi: 10.1186/s12889-021-10235-8 33478450 PMC7818563

[28] Ibrahim F, Sulaiman AI, Ahmed A. Harmful cultural traditions: An analysis of female circumcision practice in Maldives. J Advocacy, Res Educ. 2024;11(1):23–37.

[29] Shakirat GO, Alshibshoubi MA, Delia E, Hamayon A, Rutkofsky IH. An Overview of Female Genital Mutilation in Africa: Are the Women Beneficiaries or Victims?. Cureus. 2020. 10.7759/cureus.10250 PMC7536110 33042689

[30] Mitike G, Deressa W. Prevalence and associated factors of female genital mutilation among Somali refugees in eastern Ethiopia: a cross-sectional study. BMC Public Health. 2009;9:264. doi: 10.1186/1471-2458-9-264 19635149 PMC2724517

[31] Yirga WS, Kassa NA, Gebremichael MW, Aro AR. Female genital mutilation: prevalence, perceptions and effect on women’s health in Kersa district of Ethiopia. Int J Womens Health. 2012;4:45–54. doi: 10.2147/IJWH.S28805 22371659 PMC3282605

[32] Mkuwa S, Sempeho J, Kimbute O, Mushy SE, Ndjovu A, Mfaume J, et al. The role of communities and leadership in ending female genital mutilation in Tanzania: an exploratory cross-sectional qualitative study in Tanga. BMC Public Health. 2023;23(1):163. doi: 10.1186/s12889-023-15086-z 36694140 PMC9875426

[33] Ahmed HM, Kareem MS, Shabila NP, Mzori BQ. Knowledge and perspectives of female genital cutting among the local religious leaders in Erbil governorate, Iraqi Kurdistan region. Reprod Health. 2018;15(1):44. doi: 10.1186/s12978-018-0459-x 29514701 PMC5842576

[34] Asmani IL, Abdi MS. Delinking Female Genital Mutilation/ Cutting from Islam. 2008. https://www.unfpa.org/sites/default/files/pub-pdf/De-linking%20FGM%20from%20Islam%20final%20report.pdf

[35] Gambia Bureau of Statistics GB, ICF. The Gambia Demographic and Health Survey 2019-2020. Banjul: GBoS and ICF. 2021.

[36] Ahinkorah BO. Factors associated with female genital mutilation among women of reproductive age and girls aged 0–14 in Chad: a mixed-effects multilevel analysis of the 2014–2015 Chad demographic and health survey data. BMC Public Health. 2021;21(1). 10.1186/s12889-021-10293-y PMC7863379 33541311

[37] Ahinkorah BO, Hagan JE Jr, Ameyaw EK, Seidu A-A, Budu E, Sambah F, et al. Socio-economic and demographic determinants of female genital mutilation in sub-Saharan Africa: analysis of data from demographic and health surveys. Reprod Health. 2020;17(1):162. doi: 10.1186/s12978-020-01015-5 33092624 PMC7584098

[38] El-Dirani Z, Farouki L, Akl C, Ali U, Akik C, McCall SJ. Factors associated with female genital mutilation: a systematic review and synthesis of national, regional and community-based studies. BMJ Sex Reprod Health. 2022;48(3):169–78. doi: 10.1136/bmjsrh-2021-201399 35264420 PMC9279756

[39] Fikrie Z. Factors associated with perceived continuation of females’ genital mutilation among women in Ethiopia. Ethiop J Health Sci. 2011;20(1). doi: 10.4314/ejhs.v20i1.69425 PMC3275897 22434960

[40] Maalim HA. Gender issues affecting girl child education in northern Kenya. IOSR J Humanit Soc Sci. 2015;20(3).

[41] Van Bavel H. Education, Class, and Female Genital Cutting among the Samburu of Northern Kenya: Challenging the Reproduction of the “Ignorant Pastoralist” Narrative in Anticutting Campaigns. Violence Against Women. 2022;28(15–16):3742–61. doi: 10.1177/10778012221079376 35422177 PMC9619257

[42] Andiema NC. Influence of culture on girl child education in central pokot sub county, kenya. East African J Educ Stud. 2021;3(1).

[43] Mwaiko N. Overcoming obstacles to educational access for Kenyan girls: A qualitative study. J Int Womens Stud. 2017;18(2).

[44] Oyefara J. Socio-Cultural Dimensions and Attitude of Women and Community Stakeholders towards Continuation of Female Genital Mutilation (FGM) in Lagos Metropolis, Nigeria. Afr Res Rev. 2014;8(2):19. doi: 10.4314/afrrev.v8i2.2

[45] Chidera E. What factors influence the persistence of female genital mutilation in Nigeria? A systematic review. J Trop Dis. 2018;6(01).

[46] Eli S, Kalio DGB, Briggs NCT, Okagua KE. Religion, culture and medicine analysis of female genital mutilation amongst 84 antenatal clinic attendees at the Rivers State University Teaching Hospital. J Adv Med Med Res. 2020.

[47] Akweongo P, Jackson EF, Appiah-Yeboah S, Sakeah E, Phillips JF. It’s a woman’s thing: gender roles sustaining the practice of female genital mutilation among the Kassena-Nankana of northern Ghana. Reprod Health. 2021;18(1):52. doi: 10.1186/s12978-021-01085-z 33648528 PMC7923333

[48] Fosu MO, Nyarko PR, Anokye M. Female genital mutilation/cutting among Ghanaian women: The determinants. Research in Humanities and Social Sciences. 2014;4(18).

[49] Josephine U, Lydiah W. Gender based violence against females in Narok County: The dragon in their way to achieving education and safety. Int J Sci Res Publ. 2020;10(06):185–94.

[50] Mwakio NA. Overcoming obstacles to educational access for Kenyan girls: A qualitative study. J Int Womens Stud. 2017;18(2):260–74.

[51] Evelia H, Sheikh M, Njue C, Askew I. Contributing towards efforts to abandon female genital mutilation/ cutting in Kenya: A situation analysis. Popul Counc Inc. 2007.

[52] Association of Women in Media. Using the media to address FGM/ C and child marriage the case of the association of media women in. 2019.

[53] Hussein DH. Factors influencing the practice of female genital mutilation in Kenyan communities: a critical literature review. J Gend Relat Stud. 2022;3(1).

[54] Muchene KW, Mageto IG, Cheptum JJ. Knowledge and Attitude on Obstetric Effects of Female Genital Mutilation among Maasai Women in Maternity Ward at Loitokitok Sub-County Hospital, Kenya. Obstet Gynecol Int. 2018;2018:8418234. doi: 10.1155/2018/8418234 30154858 PMC6093056

[55] Esho T, Kimani S, Nyamongo I, Kimani V, Muniu S, Kigondu C, et al. The “heat” goes away: sexual disorders of married women with female genital mutilation/cutting in Kenya. Reprod Health. 2017;14(1):164. doi: 10.1186/s12978-017-0433-z 29197397 PMC5712182

